# Listening to children with obesity: lived experiences of hunger, cravings, and satiety

**DOI:** 10.1186/s12887-026-07254-y

**Published:** 2026-07-03

**Authors:** Paulina Nowicka, Nicklas Neuman, Karin Nordin, Hannah Broholm, Camilla Schollin, Ylva Törner Le Priol, Anna Ek, Karin Eli

**Affiliations:** 1 Department of Food Studies, Nutrition and Dietetics, Uppsala University, Uppsala, Sweden; 2 Department of Clinical Science, Intervention and Technology (CLINTEC), Division of Paediatrics, Karolinska Institutet, Stockholm, Sweden; 3 Warwick Medical School, University of Warwick, Coventry, United Kingdom

**Keywords:** Appetite regulation, Eating behavior, Self-regulation, Food cues, Middle childhood, Pediatric obesity treatment, Qualitative research

## Abstract

**Background:**

Appetite regulation is central to childhood obesity, yet little is known about how children themselves experience and interpret sensations of hunger, cravings, and satiety. This study aimed to explore how children living with overweight or obesity describe these sensations and how they respond to them in everyday life.

**Methods:**

This qualitative study included 32 children (17 girls, 15 boys) aged 8–10 years who were interviewed four years after enrolling in a randomized controlled obesity treatment trial in Stockholm, Sweden. At follow-up (mean age 9.4 years, SD 0.8), the majority had overweight or obesity (mean BMI z-score 2.4, SD 0.6). Individual semi-structured interviews were conducted and analyzed using thematic analysis.

**Results:**

Two overarching themes were identified. The first, *Experiences of hunger*,* cravings*,* and satiety*, comprised three subthemes: *Hunger — powerful bodily sensations*,* multiform and situational*; *Cravings — a sensory and emotional drive for something specific*; and *Satiety — recognizable bodily signals along a spectrum*. The second theme, *Responses and strategies for managing appetite*, also included three subthemes: *Self-regulation*; *External rules and structures*; and *Impulsive or emotionally driven eating*. Children described hunger as embodied and sometimes overwhelming, cravings as distinct from hunger and often cue-triggered, and satiety as ranging from comfortable to distressing fullness. Responses varied across contexts and included distraction, portion control shaped by adults, and socially motivated eating. Appetite-related experiences were typically described without moral judgment or self-blame.

**Conclusions:**

These findings position appetite regulation in middle childhood as dynamic, embodied, and context-dependent. Attending to children’s own language may inform developmentally sensitive, child-centered obesity treatment and support integration of behavioral and emerging pharmacological approaches.

## Introduction

Childhood obesity is a complex and multifactorial condition with well-documented adverse consequences for both physical and psychosocial health across the life course [1]. It is influenced by a dynamic interplay between genetic predispositions, environmental exposures, and behavioral patterns that emerge from their interaction. Among these, appetite regulation — including the sensations of hunger, cravings, and satiety — is central to understanding eating behavior [2]. Dysregulation of these sensations has been suggested to contribute to overeating, weight gain, and the persistence of obesity [3]. However, research directly addressing how children themselves perceive, interpret, and respond to these internal sensations remains limited.

While appetitive traits like hunger, cravings, and satiety are rooted in biological processes, they are experienced, interpreted, and expressed within sociocultural contexts [4]. This perspective aligns with biopsychosocial approaches to childhood eating behaviour and obesity, which emphasize how biological, psychological, and social influences interact in shaping children’s experiences of appetite and eating behaviour [5]. From a clinical perspective, this presents a challenge: physiological signals are not only sensed by the body but also understood through children’s developing cognitive and communicative capacities. In regards to this, qualitative research offers a valuable approach for exploring how appetite-related sensations are experienced and managed in everyday life [6–8]. Importantly, most of this work has been conducted on adults. In children, whose language, self-regulation, and bodily awareness are still developing, appetite-related sensations may be experienced and handled in distinct ways that warrant specific attention.

Understanding how children with obesity experience hunger, cravings, and satiety and their attempts to manage these sensations is particularly important in a clinical context, where modifying food intake is central to lifestyle treatment and where medical interventions such as GLP-1 receptor agonists (GLP-1 RAs) and bariatric surgery directly influence appetite regulation and satiety signaling [9, 10]. Children with obesity often live with heightened or persistent appetite-related challenges over time [11], yet very little is known about how they themselves make sense of and respond to such experiences in daily life. Insight into these processes is essential for developing interventions that are responsive to children’s needs and that support adaptive regulation of eating behavior without reinforcing stigma or distress.

The present study draws on follow-up data from the More and Less (ML) randomized controlled trial (Clinical trial number NCT01792531), which evaluated a parent support program for the treatment of obesity in preschool-aged children in Region Stockholm, Sweden [12, 13]. Four years after baseline, the participating children — then aged 8–10 years — were invited to take part in a qualitative interview study. This cohort has previously been the focus of several analyses: one examining social and environmental influences on eating behavior [14], and another reporting long term anthropometric and health outcomes into adolescence [15]. This cohort has also been the focus of several qualitative studies examining parents’ experiences of supporting their children’s eating and weight management following obesity treatment. In interviews conducted shortly after the intervention, parents described learning to recognize and respond to children’s hunger, cravings, and portion-related challenges, alongside efforts to establish new routines and strategies [16]. In longer-term follow-up studies, parents expressed ongoing concerns about strong hunger cues, cravings, portion sizes, and their child’s ability to regulate intake over time, as well as continued monitoring, negotiation, and anticipation in relation to eating in everyday life [17, 18]. Together, this body of work highlights the central role of parental concern and responsibility in shaping how appetite-related challenges are understood and managed within families.

The present study shifts the analytic focus to children’s own accounts of hunger, cravings, and satiety. Centering the lived experiences of children with obesity, this study aims to explore appetite regulation from the child’s perspective and to inform the development of clinically relevant, child-centered approaches to obesity prevention and treatment.

## Methods

### Study population

The participants were 32 children (17 girls, 15 boys) aged 8–10 years, all of whom had participated in the ML randomized controlled trial for obesity treatment in preschool-aged children in Region Stockholm, Sweden. The trial, conducted between 2012 and 2016, compared a structured parent support program to standard pediatric outpatient care [12]. Inclusion criteria for the trial were: children aged 4–6 years with obesity according to age- and sex-specific international cut-offs, absence of other conditions affecting growth, and parents being able to communicate in Swedish. Four years after entering the trial, these children were invited to participate in a qualitative follow-up study exploring their experiences of hunger, cravings, and satiety. As the aim was to explore children’s experiences of appetite-related sensations rather than to evaluate intervention effects, the data were analyzed across groups. Hence, the present analysis includes children from both trial arms and reflects a clinically relevant cohort with variation in treatment exposure and weight trajectories. Throughout the analytic process, attention was paid to potential differences related to trial allocation; however, no systematic patterns in children’s descriptions or strategies were identified that warranted separate analyses.

At the time of the interviews, conducted approximately four years after trial entry, the interviewed children were on average 9.4 years old (SD 0.8). 31 of the 32 children (96.9%) had overweight or obesity; including 18 children (56.3%) who met criteria for obesity. The mean BMI z-score was 2.4 (SD 0.6), indicating a clinically significant degree of excess weight. Among mothers, 15 of 30 (50.0%) had a university degree and 18 of 30 (60.0%) had a migrant background. Among fathers, 12 of 29 (41.4%) had a university degree and 15 of 29 (51.7%) had a migrant background. A more detailed description of parental characteristics has been reported previously [14].

### Data collection

Data were collected between February 2017 and December 2018 through individual, semi-structured interviews conducted by a licensed pediatric nurse with extensive clinical and qualitative research experience (KN). Interviews took place in familiar healthcare or research settings previously visited by the participants, including outpatient pediatric clinics and research facilities associated with the original trial. The interviews were arranged for the purpose of the study and were not conducted in connection with a clinical appointment. The interview guide was developed by a multidisciplinary team with expertise in child nutrition, childhood obesity, and anthropology, and covered four main areas: (1) food preferences; (2) experiences of hunger, cravings, and satiety; (3) experiences of meal situations; and (4) leisure activities. The full interview guide is provided in Appendix. As for (2), a terminological clarification is needed. Where we write “cravings,” we refer to the Swedish word *sug*. “Cravings” is the closest English equivalent but does not fully capture its meaning. *Sug* may refer to a strong urge, but it can also denote a milder feeling of temptation. When you are not hungry yet drawn (“sucked in”, as the literal translation suggests) to eat something only for the pleasure of its taste, you are “sugen”, in Swedish.

Open-ended questions were followed by prompts to encourage elaboration and reflection. Interviews lasted 11–51 min (mean 25 min), were audio-recorded, and transcribed verbatim.

### Data analysis

Transcripts were analyzed using thematic analysis, following the six phases described by Braun and Clarke [19]. The first stage involved familiarization through repeated reading of the transcripts. Open coding was conducted inductively, focusing on how children perceived and described the sensations of hunger, cravings, and satiety, as well as the strategies they used to manage these experiences. Initial coding was carried out by PN in parallel with HB and CS, allowing for comparison and refinement of codes. NN subsequently joined the analytic process and contributed to further development and consolidation of the coding framework.

Codes were iteratively collated into potential subthemes and themes through ongoing discussion and reflexive dialogue among the full research team (PN, NN, HB, CS, KN, YTLP, AE, and KE), supporting analytic rigor and credibility. The analysis emphasized children’s own perspectives and language, with representative quotations selected to illustrate each subtheme.

## Results

The thematic analysis resulted in two overarching themes that together describe children’s experiences of appetite-related sensations and how they responded to and, at times, attempted to manage these sensations in everyday life: (1) *Experiences of hunger*,* cravings*,* and satiety* and (2) *Responses and strategies for managing hunger*,* cravings*,* and satiety*. The first theme comprised three subthemes *Hunger — powerful bodily sensations*,* multiform and situational*, *Cravings — A sensory and emotional drive for something specific*, and *Satiety — recognizable bodily signals along a spectrum*. The second theme comprised three subthemes: *Self-regulation*, *External rules and structures*, and *Impulsive or emotionally driven eating*. Together, these themes capture both how children perceived and interpreted appetite-related sensations and how they acted upon them in daily life.

### Experiences of hunger, cravings, and satiety

#### Hunger — powerful bodily sensations, multiform and situational

Many children described hunger as a strong physical sensation. This was often explicitly described as being located in the stomach — as an emptiness, a rumbling, or even a sensation of pain — and sometimes accompanied by low energy or irritability. For some, it felt so clear and distinct that it was “impossible to miss.” One child explained, “When I’m hungry, it’s like my stomach says: you need food now.” Another added, “It feels empty, like something’s missing in there.” Others spoke of it as “pain” or “hurting,” or of the stomach “throbbing” when hunger became intense: “I get a bit of a stomach ache, like it’s beating a little up here [pointing towards the stomach], when I’m hungry.”

Hunger was not always experienced in the same way, however. Its intensity and character varied depending on the time of day, what the child had been doing, and how much time had passed since the last meal. “Sometimes if I haven’t eaten for several hours, I get really hungry,” said one child. “If I eat too much breakfast and then skip lunch, I get super hungry later,” noted another. Physical activity could either sharpen hunger or temporarily suppress it or distract children from the sensation of hunger. As one child noted, “If I play a lot, I don’t think about food until I stop — then I feel it all at once.”

Children also linked hunger to changes in their overall physical state or mood. Some described tiredness or a drop in energy: “My eyelids start to close — that’s when I’m running out of energy and need more.” Others pointed to emotional shifts: “I get angry, I get cranky,” said one child, while another recalled, “First I complain that I want food, and then, when I’ve been hungry for a long time, I get really mad.” In this way, hunger emerged as both a bodily and an emotional experience, shaped by context, timing, and activity. For many children, these bodily and emotional sensations were not only something to endure, but something they actively tried to manage when food was not immediately available.

#### Cravings — a sensory and emotional drive for something specific

Cravings (in Swedish: *sug*) were described as distinct from hunger. While hunger came from the body, cravings were more about desire and temptation — often triggered by smell, sight, taste, or simply thinking about a particular food. “If you smell something, and then if you feel like having it, then you really want it,” explained one child. Another put it simply: “Craving is when you really long to get something to eat.” These desires could arise even when the child was already full: “When I smell freshly baked bread, I just want it, even if I’m not hungry.”

One child explained, “If it’s something I really like, I want more even if my stomach says it’s enough.” This suggests that some children located hunger in the stomach — its epicentre — while conceptualizing cravings as arising from liking or wanting, separate from bodily need.

Unlike hunger, which could be satisfied with almost any food, cravings were often focused on a specific item, usually a favorite or something sweet. One child said “When you feel like something [specific], you want that thing.” This made cravings harder to ignore, especially if the food was nearby. “If I get a craving, it’s usually for candy or cake — not for, like, carrots,” said one child, laughing. Others gave precise examples: “I’m usually craving chocolate, candy, or something like that”, or, commenting on children in general, “Kids are usually craving pancakes.” These specific desires were often described as hard to ignore, especially if the food was nearby.

A few children described cravings as having an especially strong pull, even more intense than hunger. “If I’m craving something, I want it really badly — more than when I’m hungry,” said one child. In this way, cravings emerged not just as a milder cousin of hunger, but as their own, powerful sensory and emotional experience.

#### Satiety — recognizable bodily signals along a spectrum

Satiety was typically described as a clear feeling of fullness in the stomach, sometimes pleasant and “just right,” other times uncomfortable or even painful if the child had eaten too much. One child explained, “When I’m full, my stomach feels tight, like it’s telling me to stop.” Another shared, “Then my stomach hurts, and if I’m really full, it’s like my stomach says stop, you can’t eat more”.

Some children also described a preferred level of satiety that was neither hunger nor extreme fullness. As one child put it, “I think it’s best to be kind of in-between full.” For this child, moderate fullness was associated with feeling well and making food choices they enjoyed:

I usually take some vegetables, because I think vegetables taste really good. So if, for example, there’s corn and broccoli, I take that. And if there’s a little brown sauce, I like to take that with the corn too — I love it!

Despite this nuance, many children’s descriptions of satiety focused on the upper end of the spectrum, where fullness became uncomfortable or even distressing. For some, the sensation was linked to illness: “When I’m full it’s like having a stomach ache, not the same as when I’m hungry — more like feeling sick.” Others noted the physical heaviness: “When you’re too full, you can’t move — you just feel tired and it hurts.” One child explained why this level of fullness was particularly undesirable: “I don’t like being really, really full, because then I just feel dizzy in my head from being so full.”

A few children also described satiety as varying depending on the type of food eaten. One child explained that while fullness was usually easy to recognize with “normal food,” it could feel different with other foods: “With sushi it’s, like, kind of different. Most of the time with normal food I feel when I’m full, but with sushi you don’t really feel that you’re, like, full, even though you actually are.”

While satiety was often framed as a limit, some children linked it to positive outcomes: “It feels good, because then I can last longer and I’ve got more energy.” Together, these accounts highlight satiety not as a neutral or purely regulatory endpoint, but as a bodily state that could be experienced as pleasant, uncomfortable, or difficult to tolerate, and whose meaning varied across situations and individuals.

### Responses and strategies for managing hunger, cravings, and satiety

While the earlier theme captured feelings of hunger, cravings, and satiety, this theme describes how children dealt with these sensations in everyday life. Responses ranged from conscious self-regulation to following external rules, as well as moments of impulsive or emotionally driven eating.

#### Self-regulation

Some children described ways of managing hunger or cravings when food was not immediately available. These strategies were closely tied to their subjective sensations and involved attempts to temporarily ease or postpone feelings of hunger. Distraction was a common tactic — playing, doing sports, or watching TV to take their mind off the sensation. As one child put it, “If I feel hungry but the food isn’t ready yet, I play or do something so I don’t think about it.” Another child said: “if I’m at school I usually sit and play something with a deck of cards.” In another example, a child described physically moving away from the food trigger: “when I’m really craving popcorn and then I see the kitchen I usually just go into my room instead and try to forget that thought.” Where healthy snacks, like fruits and vegetables, were available, several children said they ate these to assuage their hunger between meals. Drinking water was also mentioned as a way to “fill up” temporarily, as one child explained: “Then my stomach feels less empty — it’s not food, but it works for a little while.”

Controlling one’s consumption when planning ahead for eating occasions was another form of self-regulation. In these cases, children spoke about managing their hunger and anticipated fullness in relation to upcoming meals or preferred foods. For example, one child described controlling his second portions of the main meal, to ensure he could eat more palatable foods later: “Sometimes I only take a little bit more, to save room if there’s going to be dessert.” Another child described managing hunger to avoid eating before a family breakfast:

Sometimes before in the morning I used to be really hungry because I wanted breakfast but I didn’t want to eat before mum and dad because I wanted to eat with them, so then I used to hold my breath, that used to help me.

Some children also described strategies they used to cope with satiety when fullness became uncomfortable. Rather than regulating intake, these strategies focused on easing bodily discomfort after eating too much. One child explained, “If I get too full, I usually just lie down.” Another described using physical activity to help the sensation pass: “If it hurts in my stomach from eating too much, I go play or ride my bike and then it goes away.” These accounts show that children not only responded to hunger and cravings, but also actively tried to manage the bodily consequences of eating beyond satiety.

#### External rules and structures

Children’s behavioral responses to hunger and appetite cues were often shaped by external rules and structures set by adults, rather than by strategies initiated by the children themselves. At home, parents decided on portion sizes and whether snacks or seconds were allowed. At school, rules could require children to try all foods served or limit how much of certain foods each child was allowed. These structures sometimes aligned with the child’s own bodily cues, but at other times constrained or overrode them. “I ask if I can have a bit more, and then they might say no or yes, depending on how much I took at the start,” one child explained. Another shared: “At school you have to take salad even if you don’t want to, otherwise you can’t leave.”

For some children, such rules helped limit eating beyond fullness – for others, they conflicted with their sense of hunger or satiety. As one child put it, in regards to the meal in school: “Sometimes I’m still hungry, but I can’t get more because there’s not enough for everyone.” As we have shown before [14], school is an important meal arena for these children, exposing them to rules and norms that are different from their homes. Faced with external rules, both within and outside of the home environment, children responded to their bodily cues through food choices allowed by parents and teachers. For example, in response to a question about whether they had “any trick… when your stomach feels empty and it rumbles,” one child explained: “We are in class then so I couldn’t do anything, no, that’s why they tell us that we can bring sandwiches or some vegetable or like fruit, I usually bring a fruit.” Another child described how her mother placed carrot pieces in the fridge, as an acceptable snack between lunch and dinner in the home: “so when I’m hungry I can just go take the bowl and eat some and when I’m full I go and put them back.”

#### Impulsive or emotionally driven eating

Some children described occasions when their responses to appetite cues were unplanned or immediate, reflecting situations where deliberate appetite regulation was not a focus. In contrast to the intentional self-regulatory efforts described above, these responses involved eating in direct response to sensory, emotional, or social cues, without attempts to manage or restrain intake.

Impulsive eating was triggered by particularly appealing food, making it hard to stop even when full. While children’s capacity for impulsive eating was limited by rules and monitoring at home and school, some children still experienced this in the context of everyday meals. As one child explained: “If it’s really tasty food, then I want to take one more serving, even though I’m actually full.” Another child related this experience to portion size: “when mum puts too much food [on my plate] and it tastes good … then I eat quite a lot anyway.”

In other cases, emotional or social situations prompted eating in the absence of hunger. One child said: “When I’m bored, I might go and look to see if there’s something nice to eat.” Another child spoke of eating when not hungry as part of conviviality:

“…like if you’re watching TV and feel like watching a movie with the family then you want something to eat even if you’re not hungry. […] then you go and watch the movie with everyone and eat together […].”

As captured in these quotes, children were aware that sensory, social, and emotional stimuli could lead them to eat in the absence of hunger. In response to these stimuli, however, children did not describe trying to manage or restrain their eating. Instead, children described this impulsive eating in largely positive terms, such as “good food”, “something nice to eat”, and “eat[ing] together”.

## Discussion

Children’s experiences of hunger, cravings, and satiety were rich and multifaceted, encompassing bodily sensations, sensory triggers, and social or situational influences. Responses to these appetite-related sensations ranged from deliberate efforts to regulate or postpone eating, to behavior shaped by external rules and structures, and to moments where appetite cues were acted upon impulsively or emotionally. The same child might, at different times, resist a craving through distraction, have their eating constrained by adult-set portion sizes, or eat beyond fullness when faced with particularly appealing foods. Together, these accounts illustrate how appetite regulation in children with obesity is dynamic and context-dependent, involving intentional self-regulation, regulation shaped by externally imposed constraints, and occasions when appetite cues are acted upon without attempts at restraint. In the paragraphs that follow, we discuss these findings in relation to previous literature on experiences of appetite sensations, children’s eating behaviors, and the future of childhood obesity treatment.

The analysis reveals that the children’s stories largely align with parental descriptions of appetite-related challenges. In an earlier analysis of interviews with parents from the same trial cohort, we found that parents were concerned about their child’s strong hunger cues and cravings, and about the difficulties these posed for managing appropriate portion sizes [16–18]. These concerns correspond closely to children’s descriptions of hunger as powerful and embodied, cravings as specific and hard to ignore, and satiety as variable and sometimes uncomfortable. At the same time, parental accounts draw attention to the background work parents described undertaking in relation to everyday eating, including monitoring, negotiating, and anticipating, which was not articulated by the children [17]. While parents described worries about impulsive eating, loss of control, and future consequences, children did not express guilt, regret, or a sense of failed self-control when describing impulsive or emotional eating. Notably, and in contrast to experiences of distress described in adult obesity research, the children in this study described impulsive or emotional eating in neutral or positive terms: as enjoyable, social, and accepted parts of everyday life. This pattern does not suggest that children dispute parents’ concerns; rather, it highlights a difference in meaning-making, with children describing appetite as a present-tense, embodied experience, while parents interpret the same behaviors through a more future-oriented lens shaped by responsibility for the child’s health and wellbeing [17].

When considering divergences between parents’ and children’s viewpoints, it is important to note that children’s accounts may reflect an earlier developmental stage, in which appetite is primarily experienced as bodily and situational, before eating becomes more strongly moralized or linked to self-evaluation [20]. Qualitative studies on children’s perspectives on living with obesity remain scarce, particularly among younger age groups, and at present there are no directly comparable studies. However, a recent Norwegian study using participatory methods with children (9–13 years) and adolescents (14–18 years) living with obesity found that older participants, particularly adolescents, more often described eating, weight, and treatment experiences in explicitly moralized or self-evaluative terms, including feelings of failure, stigma, and shame [20]. This underscores the importance of contextualizing children’s lived experiences of appetite and satiety within their developmental stage and age.

The children’s accounts did not indicate disordered eating in the form of compensatory behaviors, secretive eating accompanied by distress, or pervasive fear and guilt around food. This should be interpreted cautiously, as our interviews were not designed to assess eating disorder psychopathology and the absence of such talk is not equivalent to absence of risk. However, this observation is consistent with evidence from a systematic review and meta-analysis indicating that structured, professionally delivered pediatric obesity treatment with a dietary component is not associated with increased eating disorder risk, and may in fact be associated with reductions in eating disorder prevalence, risk, and related symptoms such as binge eating and emotional eating [21]. At the same time, some children described strategies aimed at managing hunger, cravings, or food intake. Although these were generally portrayed in neutral or positive terms and were not accompanied by self-blame or distress, it remains important for clinicians to attend to how such self-regulatory efforts are experienced by individual children and how they may evolve over time. This highlights the clinical importance of maintaining a supportive, non-stigmatizing approach to dietary change, alongside routine monitoring for emerging symptoms among children who may be more vulnerable. This interpretation is supported by recent qualitative work from the More and Less program, in which parents described feeling supported, understood, and not judged in treatment encounters, highlighting the importance of a non-stigmatizing approach for protecting children’s self-esteem and emotional wellbeing during obesity treatment [22].

Our analysis of children’s experiences of appetite and satiety as diverse, dynamic, and situational adds an important dimension to research on overeating phenotypes in children with obesity. Such research has highlighted substantial heterogeneity in appetite regulation, including differences in satiety responsiveness, food cue reactivity, emotional eating, and loss of control eating [11]. While this work has been instrumental in identifying clinically relevant subgroups using behavioral and psychometric measures, less attention has been paid to how these appetitive traits are subjectively experienced, interpreted, and managed by children in everyday life. This is particularly relevant in light of pharmacological treatments that directly target appetite regulation. A recent meta-analysis showed that GLP-1 receptor agonists significantly reduce BMI z-score and body weight in children and adolescents with obesity, including emerging evidence in children younger than 12 years [10]. These treatments are predicated on the assumption that appetite dysregulation is a key driver of obesity for at least some children, an assumption that aligns closely with the phenotype framework. However, our findings suggest that, even within a group of children living with obesity, appetitive experiences are diverse, context-dependent, and actively interpreted by children themselves. Attending to children’s own language around hunger, cravings, and satiety is therefore central to understanding how appetite-focused treatments are experienced in practice and to integrating pharmacological approaches with appropriate behavioral and communicative strategies in pediatric obesity care, including efforts to prevent adverse effects related to eating patterns.

Recent discussions around appetite-targeting treatments, including GLP-1 receptor agonists, have brought increased attention to phenomena such as food noise, a recently described construct referring to persistent or intrusive food-related thoughts [23–25], and hyperphagia, a clinical condition involving lack of satiety and constant, pathological food-seeking behavior [26]. Notably, such features were largely absent from the children’s accounts in the present study. Although children described strong hunger and cravings at times, these experiences were typically situational, fluctuating, and embedded in everyday social and emotional contexts rather than characterized by relentless food focus or abnormal food-seeking. This distinction is important in the current treatment landscape. While appetite-modulating pharmacotherapies may be highly beneficial for children with pronounced or subclinical appetite dysregulation, our findings suggest that children with obesity do not necessarily describe their appetite in ways that align with clinical definitions of hyperphagia. This highlights the importance of attending to children’s own accounts, particularly as parental or clinical interpretations of appetite may not fully correspond to how children themselves experience and articulate hunger and cravings.

Attending to how children describe and make sense of appetite may help avoid overgeneralization of concepts such as hyperphagia and food noise, and support more nuanced, individualized approaches to obesity treatment that integrate biological, behavioral, developmental, and experiential dimensions of appetite regulation. In light of the increasing use of appetite-modulating pharmacotherapies, future drug trials with children may benefit from being combined with qualitative interviews to capture how treatment effects are experienced and articulated by children at different ages. Such studies may also help identify unintended psychosocial consequences, including changes in emotional eating, food-related thoughts, or psychological wellbeing.

There are some limitations to acknowledge. The sample included children with varying treatment exposure and weight trajectories following participation in the original trial. While this heterogeneity limits conclusions regarding specific subgroups, the aim of the study was not to compare intervention effects but to explore shared and diverse experiences of appetite-related sensations in a clinically relevant population. This variation may also be considered a strength, as it captures a broader range of lived experiences among children with a history of obesity. Additionally, interviews were conducted with one parent present. This approach was chosen to maximize children’s comfort and participation given their young age and the sensitive nature of the topic. Parents did not participate in the interviews and were typically seated behind the child. Nevertheless, their presence may have influenced what children felt comfortable disclosing, particularly regarding sensitive experiences. Although weight-related stigma may influence what children feel comfortable disclosing, concerns about teasing, shame, or feeling different because of weight did not emerge spontaneously in these interviews, a finding that is consistent with our previous analysis of the same cohort. The prominence of accounts of extreme fullness may also reflect recall bias. Children living with obesity may indeed experience episodes of uncomfortable satiety more frequently; however, it is also possible that such experiences were more readily recalled or articulated, as vivid or extreme bodily sensations may be more narratively salient for children of this age. Although some children described episodes of uncomfortable fullness, the interviews did not indicate secretive eating or clandestine food consumption. Rather, children openly discussed eating in response to hunger, cravings, appealing foods, boredom, and social situations. As the study focused on children’s subjective experiences rather than detailed dietary assessment, conclusions regarding the specific behavioral mechanisms underlying persistent obesity cannot be drawn.

Finally, in developing the topic guide and conducting the interviews, we were mindful of wording questions sensitively to avoid triggering negative feelings and memories around food, eating, and the body. While this was essential to our duty of care to the participating children, it is possible that some experiences—particularly more emotionally complex ones—remained unspoken.

## Conclusions

By centering children’s own accounts of hunger, cravings, and satiety, this study highlights appetite regulation in middle childhood as an embodied and context-dependent experience rather than a stable individual trait. While previous research on childhood obesity has primarily conceptualized appetite regulation through parental reports, behavioral phenotypes, or psychometric measures, our findings show how children themselves actively interpret and manage fluctuating appetitive experiences in everyday life. Notably, these experiences were often described without the moral concern or self-blame that characterizes adult obesity narratives, and prior to the stronger moralization of eating reported in studies of older children and adolescents. Attending to children’s own language around appetite may therefore support more developmentally attuned, child-centered approaches to obesity treatment, and help align behavioral, communicative, and emerging pharmacological strategies with children’s lived experiences.

## Data Availability

The datasets generated and/or analyzed during the current study are not publicly available because they consist of qualitative interviews with children and contain information that could compromise participant privacy and confidentiality. De-identified data may be available from the corresponding author on reasonable request and subject to ethical approval and applicable data protection regulations.

## Appendix

### Interview guide

I will ask you some questions about food and meals and about what you like to do during school breaks, when you are at home, and during holidays. Some questions are also about how it can feel in your body when you are hungry or full, and when you feel that way. It is always okay to say that you do not know or that you do not want to answer. Is it okay if I ask you these questions?

My first questions are about your favorite foods. Things that you like to eat. I will also ask a few questions about foods that you do not like or that you may be unsure about.

First question: What do you like to eat?

Is there anything else that you really like to eat? If you could decide, how often would you like to eat?

Follow-up questions:

- Why do you think you cannot eat (the food mentioned above) as often as you would like?

What do you do when you are given something that you do not like?

Follow-up questions:

- Do you do the same thing at school as you do at home?

What do you do if you are given food that you have never tasted before?

Follow-up questions:

- Why do you think it can be good to try new foods?

Now I will ask some questions about how it feels to be hungry and how it feels to be full.

How does it feel in your body when you are hungry?

Follow-up questions:

- When do you usually feel like that? Every day? Before a meal? After?
- Does it always feel the same to be hungry, or can it feel different sometimes? When? How?

What do you do if you are hungry but cannot get something to eat, for example if dinner will be served soon?

Follow-up questions:

- What happens then (what do your mother, father does)?

What do you do if you are still hungry after eating one portion?

Follow-up questions:

- Do you take another portion yourself? Do you ask someone first?

What does it feel like to crave something (in Swedish: att vara sugen)? What is the difference between being hungry and craving something (in Swedish: vara hungrig och sugen)?

Do you think you think about food more or less than your classmates (or siblings)?

How does it feel in your body to be full?

When do you feel full?

Follow-up questions:

- After lunch or dinner, or do you feel that you could eat more?
- When do you usually feel that you could eat more?

Do you like the same foods as your classmates or siblings? If not, what do they like?

Follow-up questions:

- Do you think your classmates eat more than you do?
- Why do you think your friends eat more or less than you do?

Can you describe what it is like at home when you eat dinner?

Follow-up questions:

- Who do you eat with? Where do you usually sit when you eat dinner?

Now I have my last questions:

What do you like to do in your free time?

- During breaks at school?
- What do you usually do after school, in the evenings, or on weekends?
- When you are with family/siblings/friends?

## Abbreviations

BMI: Body mass index
GLP-1: Glucagon-like peptide-1
GLP-1 RA: Glucagon-like peptide-1 receptor agonist
ML: More and Less
